# Fluconazole for Hypercortisolism in Cushing’s Disease: A Case Report and Literature Review

**DOI:** 10.3389/fendo.2020.608886

**Published:** 2020-12-17

**Authors:** Yiming Zhao, Weiwei Liang, Feng Cai, Qun Wu, Yongjian Wang

**Affiliations:** ^1^Department of Endocrinology and Metabolism, The Second Affiliated Hospital Zhejiang University School of Medicine, Hangzhou, China; ^2^Department of Neurosurgery, The Second Affiliated Hospital Zhejiang University School of Medicine, Hangzhou, China

**Keywords:** Cushing’s syndrome, Cushing’s disease, fluconazole, hypercortisolism, medical treatment

## Abstract

**Background:**

Cushing’s disease is associated with an increased risk of pulmonary fungal infection, which could be a relative contraindication for pituitary adenoma excision surgery.

**Case:**

We report a case of a patient with Cushing’s disease and pulmonary *Cryptococcus neoformans*. A 48-year-old woman was admitted to our hospital because of moon face and edema. Laboratory and radiological findings suggested a diagnosis of Cushing’s disease and pulmonary cryptococcus infection. Fluconazole 400 mg per day was administered intravenously and continued orally for 3 months. Both cryptococcus infection and hypercortisolism relieved and transsphenoidal resection was performed.

**Conclusion:**

Cushing’s disease can be effectively treated with fluconazole to normalize cortisol concentration prior to pituitary surgery. Fluconazole is an alternative treatment especially in Cushing’s disease patients with cryptococcal pneumonia.

## Introduction

Cushing’s syndrome (CS) is a rare disorder caused by chronic hypercortisolism with multisystem morbidity, increased mortality and decreased quality of life (1). Surgical excision of the pituitary, adrenal or ectopic lesion is recommended as the first-line therapy of CS, but not all patients are eligible for surgery (2). Guideline recommends medical treatment in patients who are not candidates for surgery, or have recurrent disease, and in patients awaiting the effects of radiotherapy (2). Medication used in patients with CS can be classified into steroidogenesis inhibitors, pituitary-targeting agents, and glucocorticoid receptor antagonists (3). Steroidogenesis inhibitors can block various steps of the steroid synthesis pathway. Ketoconazole, the most widely used medication, is unavailable in most countries and regions because of the risk of severe hepatotoxicity (4). New steroidogenesis inhibitors, including osilodrostat and levoketoconazole, have shown efficacy and acceptable safety profiles in clinical trials (5, 6). Though osilodrostat is approved by US Food and Drug Administration (FDA), it has not been used widely due to unavailability (7). The present work demonstrates the effect of fluconazole in controlling hypercortisolism in a patient with Cushing’s disease (CD) and pulmonary cryptococcus infection. All cases previously reported in the literature are also reviewed. In addition, a comprehensive bioinformatics analysis has been done to identify the potential targets of fluconazole in CD.

## Case Presentation

A 48-year-old woman visited the hospital for evaluation of facial swelling and fatigue lasting for more than one year. She reported 5 kg of weight gain, hypertension, insomnia, weakness, and easy bruising. She denied any fever, oligouria, chest distress or shortness of breath. She went to the local hospital and the examinations were unremarkable except “hypokalemia.” She had been treated with irbesartan at a daily dose of 150 mg for 1 year. The social history and family history was unremarkable.

On examination, the blood pressure was 164/84 mm Hg, the pulse 56 beats per minute, the weight 53.7 kg, the height 146 cm and the body mass index (BMI) 25.19 kg/m^2^. She had moon face, dorsal fat pad, abdominal obesity and ecchymosis, but no striae. The remainder of the examination was normal.

**Figure 1:**
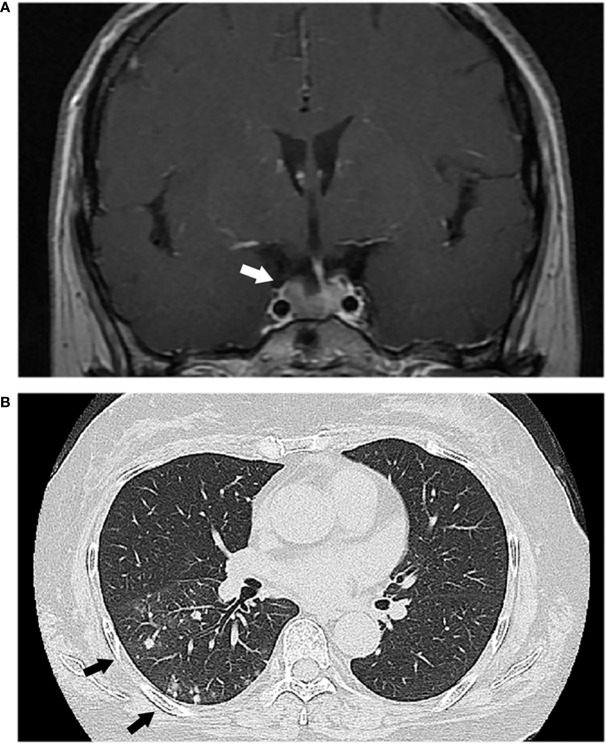
**(A)** Magnetic resonance imaging showed a pituitary adenoma (white arrow). **(B)** Chest computed tomography revealed multiple lung nodules (black arrows).

Initial investigations were summarized in **Table 1**. A midnight serum cortisol was 16.2 ng/dl and adrenocorticotropic hormone (ACTH) was 94.9 pg/ml 24-h urinary free cortisol (UFC) was 813.5 μg/day. Cortisol failed to suppress during a 48-h low-dose dexamethasone suppression test (16.1 ng/dl). These findings were consistent with an ACTH-dependent CS. No suppression was seen with high-dose dexamethasone test. A pituitary magnetic resonance imaging (MRI) demonstrated a 1.2 cm adenoma (**Figure 1A**). Inferior petrosal sinus (IPS) sampling confirmed a pituitary source of ACTH secretion. At the same time, the chest computed tomography revealed multiple lung nodules (**Figure 1B**). Bronchoalveolar lavage fluid (BALF) and serum cryptococcal antigen was positive. Lung biopsy histology confirmed pulmonary *Cryptococcus neoformans*. Cerebrospinal fluid (CSF) cryptococcal antigen was negative.

**Table 1:** Laboratory data.

Variables	Reference Range, Adults	At admission	Follow up (3 months after admission)	Follow up (3 months after neurosurgery)
24-h UFC (µg)	28.5–213.7	813.5	42.2	NA
8:00 am cortisol (µg/dl)	6.7–22.5	17.0	1.4	18.7
4:00 pm cortisol (µg/dl)		23.2	1.2	11.3
Midnight cortisol (µg/dl)		16.2	NA	NA
8:00 am ACTH (pg/ml)	7.2–63.3	85.9	53.2	56.9
4:00 pm ACTH (pg/ml)		109.1	43.3	33.9
Midnight ACTH(pg/ml)		94.9	NA	NA
Potassium (mmol/L)	3.5–5.5	3.34	3.52	4.24
ALT (U/L)	<35	23	21	NA
AST (U/L)	<35	22	22	NA
Aldosterone (pg/ml)	30–353	<30	NA	NA
Renin (uIU/ml)	4.4–46.1	11.6	NA	NA
Testosterone (nmol/L)	<2.53	1.88	1.52	NA
Low-dose dexamethasone suppression test cortisol (µg/dl)	<1.8	16.1	NA	NA
High-dose dexamethasone suppression test cortisol (µg/dl)		9.9	NA	NA
% Change from baseline	Expect >50% suppression for a positive test for Cushing’s disease	41.9	NA	NA
IPS: peripheral ACTH ratio	Expect >2.0 in Cushing’s disease	3.4	NA	NA
IPS: peripheral prolactin ratio	Expect >1.8 in successful catheterization	2.9	NA	NA

UFC, urinary free cortisol; ACTH, adrenocorticotropic hormone; AST, aspartate transaminase; ALT, alanine aminotransferase; IPS, Inferior petrosal sinus; NA, not available.

Immediate transsphenoidal selective adenomectomy (TSS) was not an option due to cryptococcus infection and possible postoperative cryptococcal meningitis. Intravenous fluconazole was started with 400 mg daily. One week later, the patient was discharged and continued with oral fluconazole 400 mg per day. At 3 months follow-up the pulmonary infection improved and the UFC was 42.2 μg/day. Fluconazole was withdrawn and TSS was performed. Histopathology confirmed ACTH-secreting pituitary adenoma.

Three months after TSS, the patient was in good general health and her serum cortisol and UFC normalized.

## Discussion

TSS performed by an experienced neurosurgeon is the first-line treatment for CD in most patients (2). Patients with CD have an increased risk of cyrptococcal infection (8). Undiagnosed cryptococcal pneumonia can lead to postoperative cryptococcal meningitis and poor prognosis (9). Medical therapy is needed in patients with hypercortisolism when surgery is not possible. Ketoconazole, which is used to control both hypercortisolism and fungal infections, is unavailable due to severe adverse effect. Fluconazole, another azole antifungal, is an alternative to ketoconazole with less hepatotoxicity.

The present work revealed only 5 other CS patients treated successfully by fluconazole when surgery was not possible or was noncurative (as showed in **Table 2**) (10–14). All of patients were female, which could be attributed to the female predominant prevalence of CS (female-to-male ratio 3:1) (1). Three of them were diagnosed as CD, two with ectopic ATCH-secreting syndrome (EAS), and one had adrenal carcinoma. The median UFC levels was 310 µg/day (range, 112–813.45 µg/day). Hypocortisolism was more prone to occur in CD patients instead of other kinds of CS. The reported dose of fluconazole ranged from 200 to 1200 mg/day. Two of them developed liver dysfunction with the daily dose of fluconazole more than 400 mg. Liver enzymes normalized after the dose decreased to 400 mg/day.

**Table 2:** Clinical features of 6 patients with CS successfully treated with fluconazole.

Patient	Age	Race	CS	24 h UFC (µg)	ACTH (pg/ml)	Serum cortisol (µg/dl)	Comorbidities	Treatment of CS	Dose of fluconazole	AE	FU 24 h UFC (µg)	Outcome
1 (our patient)	48	Asian	CD	813.5	85.9	17.1	Cryptococcal pneumonia	Fluconazole and pituitary adenoma excision	400 mg QD	NA	42.2	Relieved
2 (10)	83	NA	Adrenal carcinoma	295	NA	19.4	Sepsis	Adrenalectomy and fluconazole	200–400 mg QD	NA	8.4	Stable
3 (11)	61	Asian	CD	273	99.0	34.0	NA	Pituitary adenoma excision, metyrapone, ketoconazole and fluconazole	400 mg QD	NA	Normal	Stable
4 (12)	50	Asian	CD	325	NA	27.5	Hypertensive basal ganglia hemorrhage	Ketoconazole, cabergoline and fluconazole	400 mg QD	NA	56	Relieved
5 (13)	39	NA	EAS	775	572.9	175.8	Gastrointestinal tract bleeding	Fluconazole and bilateral adrenalectomy	400–600–400 mg QD	Increased liver enzyme levels	NA	Relieved
6 (14)	80	NA	EAS	112	378.0	6.0	Myelodysplasia	Fluconazole	200–1,200–400 mg QD	Increased liver enzyme levels	NA*	Died

*Normalization of cortisol levels.

CS, Cushing’s syndrome; UFC, urinary free cortisol; ACTH, adrenocorticotropic hormone; AE, adverse effect; FU, follow-up; CD = Cushing’s disease; EAS, ectopic ATCH-secreting syndrome; NA, not available.

In previous reports, inhibitory effect of fluconazole on glucocorticoid production was controversial and less potent than ketoconazole (10, 15, 16). Some case reports showed adrenal dysfunction with fluconazole in patients with server comorbidities (17–20). However, another study suggested that fluconazole was not associated with adrenal insufficiency compared with placebo (21).

The different in vitro effect of fluconazole could be attributed to the different experimental cell lines and different expression of steroid synthesizing enzymes of the cells. Previous studies demonstrated potent inhibitory effect of fluconazole on cortisol production in several human cell culture lines, but only a weak effect in rat cell culture lines (10, 15, 16). A previous study has revealed that the polymorphism in the CYP17A1 gene is associated with the responsiveness to steroidogenesis inhibitors in CS patients (22). However, the relationship of genetic variants with the efficacy of fluconazole is unknown.

**Figure 2:**
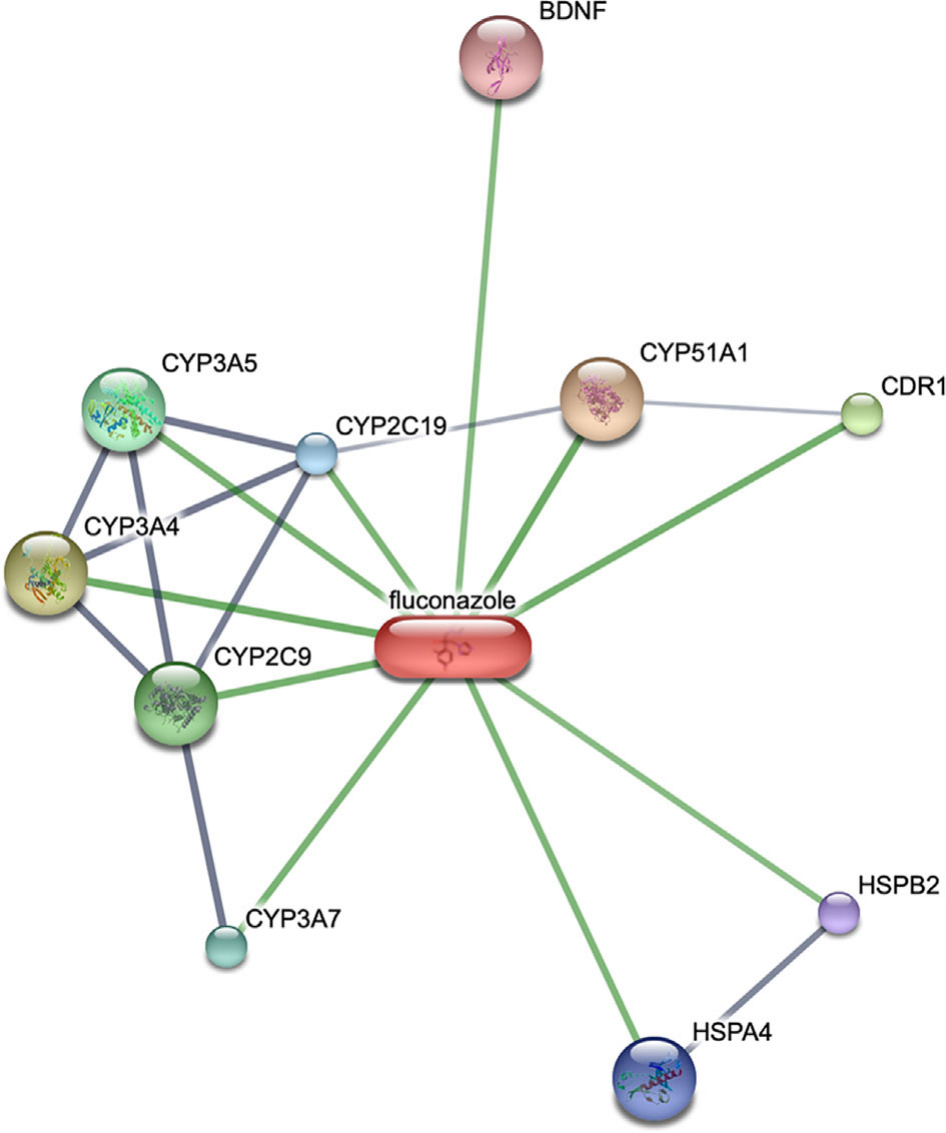
Potential targets of fluconazole and Cushing’s disease from STITCH. The node size reflects the degree of relationship: the smaller the degree value, the smaller the node size is.

We predict potential targets of fluconazole and obtained a network from STITCH (**Figure 2**). STITCH (http://stitch.embl.de) is a unique system pharmacological database describing the relationships between drugs, targets, and diseases (23). The results showed brain-derived neurotrophic factor (BDNF) was a target of fluconazole. BDNF is the neurotrophin mediating pro-neuronal survival and plasticity. Fiocco’s study showed BDNF regulated the activity of hypothalamic-pituitary-adrenal axis (24). Similar studies showed BDNF polymorphism had an influence on individual cortisol response to stress (25, 26). In Cushing disease patients, decreased BDNF level was observed after remission indicating a potential role of BDNF in Cushing disease (27). Over the past two decades, studies showed that BDNF and its receptor tropomyosin receptor kinase B (TrkB) were up-regulated in many types of cancers such as breast cancer and cervical cancer (28–30). The activated BDNF/TrkB signal stimulates a series of downstream pathways, including phosphoinositide 3-kinase/protein kinase (PI3K), Ras-Raf-mitogen activated protein kinase kinase-extracellular signal-regulated kinases, the phospholipase-C-γ pathway and the transactivation of epidermal growth factor receptor (28). Dworakowska’s study showed PI3K pathway was upregulated in ACTH- pituitary adenomas (31). Song’s study showed PI3K/AKT signaling pathway could affect migration and invasion of pituitary adenoma (32). In this series, two patients with fluconazole therapy showed decreased ACTH levers, which could not be attributed to effects on adrenocortical steroidogenesis (14). We propose the following hypothesis, BDNF/TrkB and PI3K pathways are potent therapeutic mechanisms for fluconazole in Cushing disease. In the future, more in vitro and in vivo studies are needed to verify our findings.

## Concluding Remarks

In summary, this case report and the bioinformatics analysis suggest that fluconazole might be effective in controlling hypercorticolism in CD patients. Further studies on the mechanism by which fluconazole inhibits cortisol production are needed to develop more potent and less toxic agents.

## Data Availability Statement

The original contributions presented in the study are included in the article/supplementary material. Further inquiries can be directed to the corresponding author.

## Ethics Statement

The patient provided written informed consent for research participation as well as for the publication of indirectly identifiable data (age, gender, and medical history).

## Author Contributions

YZ and WL wrote the first draft of the manuscript. YZ, QW, FC, and YW made contributions to the acquisition of the clinical data. YZ and YW made critical revisions and approved final version. All authors contributed to the article and approved the submitted version.

## Funding

This study was supported by the Natural Science Foundation of Zhejiang Province (LQ19H160024).

## Conflict of Interest

The authors declare that the research was conducted in the absence of any commercial or financial relationships that could be construed as a potential conflict of interest.
